# Genome-wide association mapping in exotic × Canadian elite crosses: mining beneficial alleles for agronomic and seed composition traits in soybean

**DOI:** 10.3389/fpls.2024.1490767

**Published:** 2024-11-14

**Authors:** Katherine Fortune, Sepideh Torabi, Milad Eskandari

**Affiliations:** Department of Plant Agriculture, University of Guelph, Plant Agriculture, Guelph, ON, Canada

**Keywords:** soybean, nested association mapping, yield, MTAS, GWAS, exotic lines, enhancing germplasm

## Abstract

Given the narrow genetic base of North American soybean germplasm, which originates from approximately 35 ancestral lines, discovering and introducing useful diversity for key traits in exotic germplasm could potentially enhance diversity in the current elite gene pool. This study explores the potential of exotic germplasm to enhance yield and agronomic traits in the University of Guelph soybean germplasm. We utilized a nested association mapping (NAM) design to develop a population (n = 294) composed of crosses of high-yielding Canadian elite cultivar, OAC Bruton, with four high-yielding exotic lines developed at USDA (Urbana, IL), and we mapped the genetic architecture of agronomic and seed composition traits using association mapping methods. The analysis across three Southwestern Ontario environments revealed seven unique genomic regions underlying agronomic traits and four for seed composition traits, with both desirable and undesirable alleles from the exotic parents. Notably, a region on chromosome 10, co-locating to the *E2* maturity locus, was found to be associated with seed yield and maturity. The allele that increased yield by 166 kg/ha was contributed by all exotic parents and was absent in the Canadian-adapted parent. The study underscores the potential of using exotic germplasm to introduce novel genetic diversity into the Canadian elite soybean breeding pool. By identifying exotic-derived beneficial alleles, our findings offer a pathway for enhancing agronomic traits in Canadian soybeans with novel exotic diversity.

## 1 Introduction

Soybean is the fourth largest crop in Canada by production area, with nearly two-thirds of the harvest destined for export (Soy Canada, 2024). To match the increasing global demand for soybean, Soy Canada aims to increase annual national food-grade soybean production by 25% (1.8 tonnes) by 2030. Soybean acreage is expected to grow as production rapidly expands into the western prairies. However, plant breeding efforts in developing high-yielding cultivars will be crucial to addressing increased production demands (Soy Canada, 2022).

Soybean has seen notable yield improvement in North America in the past 100 years. Bruce et al. (2019a) summarized several previous yield studies and reported that annual yield increases due to genetics ranged from 11 to approximately 32 kg/ha/year in germplasm from Maturity Group (MG) IV to 000. In the context of Canadian MG 2 to 0, the yield increase ranges from 15.7 to 16.1 kg/ha/year relative to historical lines (Bruce et al., 2019a). However, yield improvement is set against the backdrop of a narrow genetic base of North American soybeans, with only 35 ancestral lines [plant introductions (PIs) and landraces] contributing over 95% of all alleles (Gizlice et al., 1994).

The constraint of genetic diversity raises concerns about the long-term sustainability of yield improvements. While modern soybean breeding has successfully exploited a limited genetic base without significant erosion (Bruce et al., 2019b; Hyten et al., 2006), evolving climate, pests, and diseases and increasing demand for crop productivity necessitate a broader genetic toolkit (McCouch et al., 2013). Incorporating useful genetic diversity into elite breeding pools is a proactive strategy to increase the yield, adaptability, and resilience of future soybean cultivars (Kofsky et al., 2018).

Seed yield is the most important agronomic trait, but understanding its genetics is difficult because it is a complex quantitative trait. Yield improvement largely depends on yield component traits and tightly correlated traits such as plant architecture, phenology, and resistance to biotic and abiotic stresses, which protect yield potential. Phenology highly correlates with yield potential, such that high yield is positively correlated with later maturity in soybean (Diers et al., 2018). Several studies based on the SoyNAM project (http://www.soybase.org/SoyNAM) highlighted the efficacy of the utilization of nested association mapping (NAM) designs for exploring genetic architectures and assessing the contributions of exotic parents for key quantitative traits. Agronomic, seed composition, water use efficiency, and quantitative disease resistance are some key traits that have been dissected in the SoyNAM project (Diers et al., 2023, 2018; Lopez et al., 2019; Scott et al., 2019). For example, using the SoyNAM population, Diers et al. (2018) identified a large effect of exotic-based yield quantitative trait locus (QTL) on chromosome 8 from exotic lines with PI ancestry, demonstrating the usefulness of mining useful diversity in exotic germplasm.

The NAM population design in plant crops was originally created by maize geneticists to harness the strengths of both traditional bi-parental linkage mapping and association mapping in one population for dissecting complex quantitative traits (Yu et al., 2008). A common “hub” parent is crossed to a set of diverse founder parents to create a half-sibling structured recombinant inbred line (RIL) mapping population, which has more allelic diversity than bi-parental mapping populations, but less cofounding population structure compared to a genome-wide association study (GWAS) panel (Gage et al., 2020; Scott et al., 2020; Yu et al., 2008). In NAM populations, the genetic structure is shaped by both historical and recent recombination events among different parental lines. This leads to larger haplotype blocks and extended linkage disequilibrium (LD), reducing the need for dense markers to capture parental haplotypes compared to traditional GWAS panels (Song et al., 2017).

In this study, a panel of 294 RILs derived from four bi-parental cross combinations of Canadian elite × exotic lines were assessed across three environments in Southwestern Ontario. The objectives of this study were to 1) characterize the genetic diversity among the parental lines chosen for the population, 2) identify genomic regions associated with agronomic and seed composition traits, and 3) identify alleles from exotic accessions that can be used to improve agronomic traits in Canadian germplasm.

## 2 Materials and methods

### 2.1 Population development and phenotyping

The population, composed of 294 F4-derived RILs, was constructed by crossing four wild-derived experimental lines to the common Canadian elite cultivar, OAC Bruton (Torabi et al., 2018). OAC Bruton is an indeterminate, high-yielding, large-seeded, soybean cyst nematode (SCN)-resistant food-grade soybean cultivar adapted to MG 1 to 2 in southern Ontario, Canada, developed by the University of Guelph, Ridgetown program. The wild soybean-derived experimental lines (LG lines) are LG14-13101, LG15-1913, LG15-2959, and LG15-4075. These lines are high-yielding exotic experimental lines with MG 2 to 3 rating developed by the US Department of Agriculture (USDA) Agricultural Research Service (ARS) in Urbana, IL (Project : USDA ARS, 2024) (**Table 1**).

**Table 1:** Pedigree, percentage of plant introduction (PI) ancestry, and characteristics of common parent, OAC Bruton, and the four exotic lines, LG14-13101, LG15-1913, LG15-2959, and LG15-4075, used to construct the NAM population.

NAM family	Parent	Pedigree of parent	Origin	% PI	Characteristics
	OAC Bruton	Cultivar: SC Starfield × SC 2307	University of Guelph, Ridgetown Campus		RM 1.8, high yield, protein, and oil, large-seeded, SCN resistant
138 (OAC Bruton × LG14-13101)	LG14-13101	BC3 PI 441001 × Dwight (PI 597386)	USDA –ARS (Urbana, IL)		RM 2.0, high yield, moderate protein and oil, diverse ancestry
139 (OAC Bruton × LG15-1913)	LG15-1913	F3:5 LG10-2695 × LD09-30015	USDA– ARS (Urbana, IL)	16	RM 2.0, high yield, moderate protein and oil, diverse ancestry,
140 (OAC Bruton × LG15-2595)	LG15-2595	F3:5 LG11-6190 × LD09-30015	USDA– ARS (Urbana, IL)	28	RM 3.0, high yield, moderate protein and oil, diverse ancestry
141 (OAC Bruton × LG15-4075)	LG15-4075	F3:5 LD09-30015 × LG09-7739	USDA– ARS (Urbana, IL)	38	RM 3.0, high yield, moderate protein and oil, diverse ancestry

NAM, nested association mapping; SCN, soybean cyst nematode.

The RIL populations along with their respective parents were planted in three test environments over 2 years in Southwestern Ontario, Canada. The test environments included Chatham (42°23′58.5″N 82°07′17.1″W) and Palmyra (42°25′50.1″N 81°45′06.9″W) in 2022 and Ridgetown (42°27′14.8″N 81°52′48.0″W) in 2023. A randomized complete block design (RCBD) with two replicates was used in each field-testing location, where each plot consisted of five rows, 4.2 m long, with a row spacing of 43 cm. A total of 500 seeds were planted in each plot to achieve a plant density of 54 seeds per m^2^. The rows were trimmed to 3.8 m in length after emergence, and the middle three rows were machine harvested at maturity for estimating yield performance and measuring seed quality traits.

The traits of interest in this study were seed yield (SY), plant height (PH), days to maturity (DTM), seed weight (SW), and seed composition traits, which included the seed concentration of protein, oil, and sucrose on a dry seed basis. The agronomic traits including DTM and PH were scored in the field at maturity. DTM was scored as days from planting to stage R8 (95% pods fully mature), and PH was measured as the distance from soil level to the top node on the main stem in centimeters. DTM and PH data were not collected in the Ridgetown 2023 environment. Seed traits including SY, SW, and seed composition traits were measured from the harvested three middle rows of seeds. SY and SW were measured for each harvested plot along with seed moisture, adjusted to 13% moisture. SY was estimated on a kg/ha basis, and SW was measured as the weight of 100 seeds (in grams). Seed composition traits were determined based on subsampling the total harvested plots on a dry basis (0% seed moisture) and measured as an average of three technical replicate readings. The seed composition traits were measured as the percentage of dry seed weight using a Perten DA 7250 SD near-infrared reflectance (NIR) analyzer (Perten Instruments Canada, Winnipeg, MB, Canada).

### 2.2 Statistical analysis

Analysis of variance (ANOVA) was conducted to obtain the best linear unbiased estimators (BLUEs) of genotypes for all traits. The models were fitted according to Equation 1 using the lme4 package (v1.1.35.1, Bates et al., 2015) in R statistical software version 4.2.3.


(1) $$y_{ijk}=\mu +E_{i}+G_{j}+{\left(G\times E\right)}_{ij}\;+B_{k\left(i\right)}+e_{ijk}$$

where $y_{ijk}$ is the observed trait value; μ is the overall mean (μ); $E_{i}$ , $G_{j}$ , and ${\left(G\times E\right)}_{ij}\;$ are the fixed environment, genotype, and environment–genotype interaction, respectively; $B_{k\left(i\right)}$ is the random nested block effects within environments; $e_{ijk}$ is the residual error.

Analysis of covariance (ANCOVA) was conducted to test the significance of maturity on yield and plant height according to Equation 2, and the full and reduced models were compared using the Akaike information criterion (AIC) statistic.


(2) $$y_{ijk}=\mu +X+E_{i}+G_{j}+{\left(G\times E\right)}_{ij}+B_{k\left(i\right)}+e_{ijk}\;$$

where $y_{ijk}$ is the observed trait value; μ is the overall mean (μ); X is the vector of days to R8 physiological maturity treated as a fixed effect; $E_{i}$ , $G_{j}$ , and ${\left(G\times E\right)}_{ij}$ are the fixed environment, genotype, and environment–genotype interaction; $B_{k\left(i\right)}$ is the random nested block effects within environments; $e_{ijk}$ is the residual error.

Broad-sense heritability ( $H^{2}$ ) was calculated within each RIL family as well as across the entire NAM population on an entry means basis according to Holland et al. (2002). The $H^{2}$ for each of the four RIL families was calculated according to Equation 3. Additive single-nucleotide polymorphism (SNP) heritability was also estimated using a linear mixed model implemented in Genome-Wide Complex Trait Analysis (GCTA) (Yang et al., 2010, 2011).


(3) $$H^{2}=\;\frac{\sigma_{RIL{\left(family\right)}_{p}}^{2}}{\sigma_{RIL{\left(family\right)}_{p}}^{2}+\;\frac{\sigma_{env*RIL{\left(family\right)}_{p}}^{2}}{n_{env}}+\frac{\sigma_{\epsilon }^{2}}{n_{plot}}\;}$$

where $\sigma_{RIL\left(family\right)}^{2}$ is the variance among RILs nested in the $p^{th}$ family. $H^{2}$ across the entire population was calculated from a variance model with RILs nested within families (Equation 4) and calculated according to Equation 5.


(4) $$\begin{array}{c} y_{ijk}=\mu +E_{i}+F_{j}+{\left(F\times E\right)}_{ij}+{\left(F\left(G\right)\times E\right)}_{ijl}+G_{l\left(j\right)}+\;B_{k\left(i\right)}\\ +e_{ijkl} \end{array}$$

where $y_{ijk}$ is the observed trait value, μ is the overall mean (μ), $E_{i}$ is the random environment effect, $G_{j}$ is the random family effect, ${\left(F\times E\right)}_{ij}\;$ is the family–environment interaction, ${\left(F\left(G\right)\times E\right)}_{ijl}$ is the RILs nested in family × environment interaction, $B_{k\left(i\right)}$ is the nested block effects within environments, and $e_{ijk}$ residual error.


(5) $$H^{2}=\frac{\sigma_{family}^{2}+\frac{1}{4}\sum_{p=1}^{4}=\sigma_{RIL{\left(family\right)}_{p}}^{2}}{\sigma_{family}^{2}+\frac{1}{4}\sum_{p=1}^{4}=\sigma_{RIL{\left(family\right)}_{p}}^{2}+\frac{\sigma_{env*family}^{2}}{n_{env}}+\frac{\sigma_{env*RIL(family)}^{2}}{n_{env}}+\frac{\sigma_{\epsilon }^{2}}{n_{plot}}}$$

where $\sigma_{family}^{2}$ is the variance among families and $\sigma_{RIL{\left(family\right)}_{p}}^{2}$ is the variance among RILs nested in the $p^{th}$ family.

### 2.3 Genotyping and SNP analysis

Fresh tissue from young trifoliate leaves was collected from the first replicate in the Chatham 2022 field trial (F_6_ generation) and freeze-dried for 48 hours using a Savant ModulyoD Thermoquest (Savant Instruments, Holbrook, NY, USA). High-quality DNA extraction was conducted from tissue samples using the Macherey Nagel NucleoSpin Plant II DNA miniprep kit and protocol (MACHEREY-NAGEL, Düren, Germany). DNA quantity and quality were checked using a NanoDrop spectrophotometer (Thermo Fisher Scientific, Waltham, MA, USA), and all DNA samples were standardized to a final concentration of 20 ng/µL. Samples with 10-µL volumes were sent to Plate-forme D’analyses Genomique at Laval University for genotyping by sequencing (GBS) based on PE150 (pair-ended) library prep via *Bfa*I restriction enzyme digest (Torkamaneh et al., 2021) and sequenced on a single lane of Illumina NovaSeq 6000.

A total of 294 RILs and the five parental lines (N = 299) were genotyped; however, 46 RILs were removed in the bioinformatics pipeline due to low sequencing depth. The remaining population size for the genetic study was 253 RILs. The sample size per population was 66 for population 138 (OAC Bruton × LG14-13101), 72 for population 139 (OAC Bruton × LG15-1913), 42 for population 140 (OAC Bruton × LG15-2959), and 63 for population 141 (OAC Bruton × LG15-4075). The Fast-GBS v2 reference-based pipeline (Torkamaneh et al., 2020) was used to call SNPs. Briefly, the FASTQ files were demultiplexed, trimmed, and aligned to the Gmax_275_v2 reference genome, and SNP variants were called using the open-source command-line tools Sabre, Cutadapt, BWA, and Platypus (Martin, 2011; Li, 2011; Rimmer et al., 2014). Missing genotype data were imputed using Beagle v5.1 (Browning and Browning, 2016). Of the 294,279 variants called from the FastGBS pipeline, 5,213 remained after imputation and quality control filtering. SNP variants were filtered out if i) they were multi-allelic, ii) missing data > 15%, iii) heterozygosity > 15%, iv) minor allele frequency (MAF) >0.05, and v) non- polymorphic between the five parental lines.

A total of 5,215 SNP markers were used to measure LD in the population using the PopLDdecay command line tool (Zhang et al., 2019). Pairwise correlations between every marker at a maximum distance of 8,000 kb were calculated, and the mean r^2^ for each 100 kb and 1,000 kb was plotted for the heterochromatin and euchromatin regions. A trend line was fitted using a LOESS function, and LD decay distance was estimated at the threshold of r^2^ = 0.2. A neighbor-joining tree based on identity by state (IBS) distances was constructed to visualize the genetic relationship among the five parental lines using TASSEL v.5 and visualized using the Interactive Tree of Life website (Letunic and Bork, 2016). To visualize the population structure in the population, principal component (PC) analysis was conducted in TASSEL using the 5,213 SNP markers. The number of PCs that capture the most variation in the population was determined using a scree plot that plots the eigenvalues of each PC.

### 2.4 Nested association mapping

The association mapping was conducted using the package NAM (Xavier et al., 2015) developed for the analysis of multi-parental populations such as the NAM design. This method is designed to account for the genetic structure in NAM populations by using subpopulation to define the stratification factor.

The mixed linear model used by this method for GWAS is


y=μ+Xb+ɡ+e 

where *y* is the vector of observed phenotypes, *µ* is the overall mean, *X* is the allele matrix from SNP data informed by subpopulation stratification factor, *b* is the vector of the fixed SNP effect within subpopulations, *ɡ* is the polygenic term estimated from kinship matrix, and *e* is the error variance.

The average allele effect across all families as opposed to the family-specific effects was reported due to the relatively small and uneven sample sizes of each family, which may overestimate effect size. To deal with multiple testing in the association mapping, a false discovery rate (FDR) threshold at *α* ≤ 0.05 level [−log10(p-value) = 3.8] was used to declare SNP significance. GWAS analyses were separately conducted for individual and combined environments to detect marker-trait associations (MTAs) that were stable across environments, considering the existence of traits’ interaction with the environment (G×E interaction) (Malosetti et al., 2013).

## 3 Results

### 3.1 Phenotypic variation

**Figure 1:**
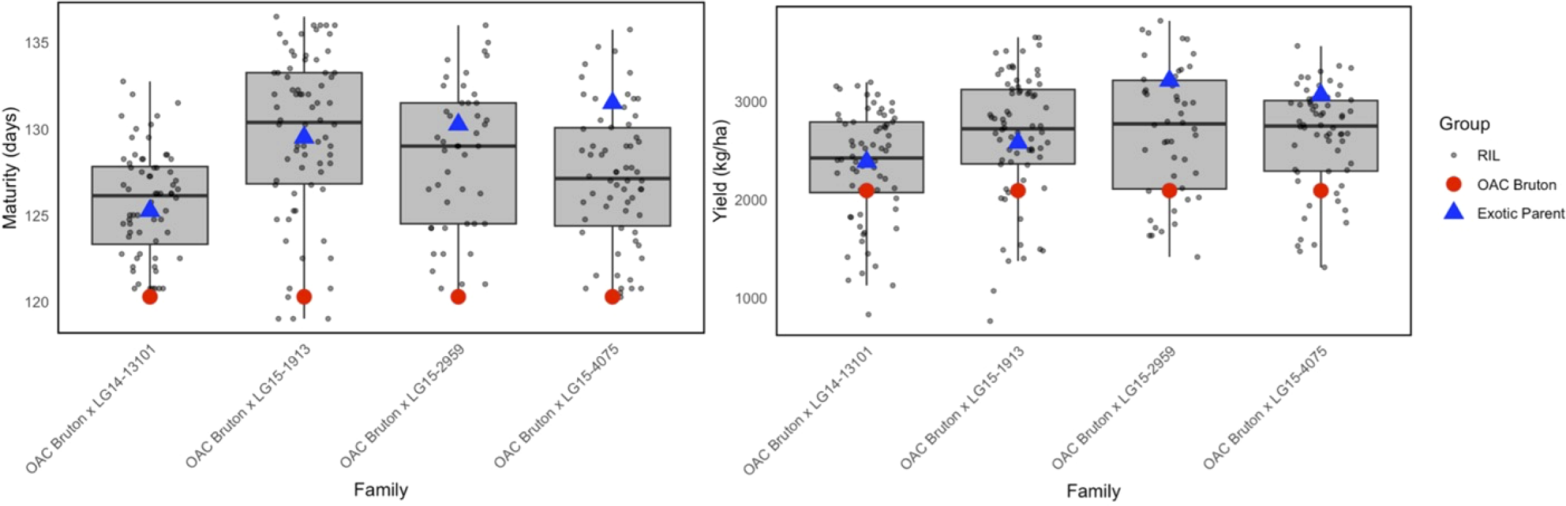
Boxplots of best linear unbiased estimate (BLUE) values for maturity and yield in four families created from crossing elite OAC Bruton (red) to four exotic lines: LG14-13101, LG15-1913, LG15-2959, and LG15-4075. The box spans the interquartile range in which 50% of recombinant inbred line (RIL) values lie, and the horizontal line in each box represents median value of RILs.

In the combined environment analysis, exotic parents matured 9 days later than OAC Bruton on average, and they produced more SY than OAC Bruton (**Figure 1**). ANCOVA was conducted to test the significance of maturity on yield and plant height. Maturity had a significant effect on SY and PH and was therefore included as a covariate to adjust the effect of genotype for maturity effects (**Supplementary Table 1**).

The distribution of all traits across environments is shown in **Supplementary Figure 1** and **Supplementary Table 2**. Significant genotype variation was observed for all measured traits in the NAM population, and analyses of variances revealed genotype and G×E interaction as the main sources of phenotypic variances (**Supplementary Table 3**). Across families and combined environments, $H^{2}$ ranged from 0.59 to 0.73 for SY, 0.82 to 0.90 for DTM, 0.18 to 0.66 for PH, 0.72 to 0.80 for SW, 0.74 to 0.89 for PRO, 0.74 to 0.84 for OIL, and 0.13 to 0.60 for SUC (**Table 2**). SNP-based heritability is useful for estimating the proportion of phenotypic variation attributed to the additive genetic variation of SNPs. SNP-based heritability estimates are consistently lower than broad-sense heritability across traits, indicating that the SNPs capture only a portion of total genetic variance.

**Table 2:** Broad-sense heritability and SNP-based heritability estimates of traits across all populations.

Trait	Population					
	138	139	140	141	All	SNP-H^2^
Yield	0.628	0.718	0.592	0.734	0.448	0.331
Protein	0.799	0.740	0.794	0.888	0.825	0.389
Oil	0.801	0.744	0.843	0.792	0.431	0.358
Sucrose	0.448	0.598	0.128	0.593	0.339	0.366
Seed weight	0.800	0.768	0.737	0.724	0.702	0.343
Plant height	0.656	0.563	0.182	0.487	0.264	0.136
Maturity	0.815	0.900	0.873	0.882	0.622	0.211

SNP, single-nucleotide polymorphism.

### 3.2 Linkage disequilibrium and population structure

**Figure 2:**
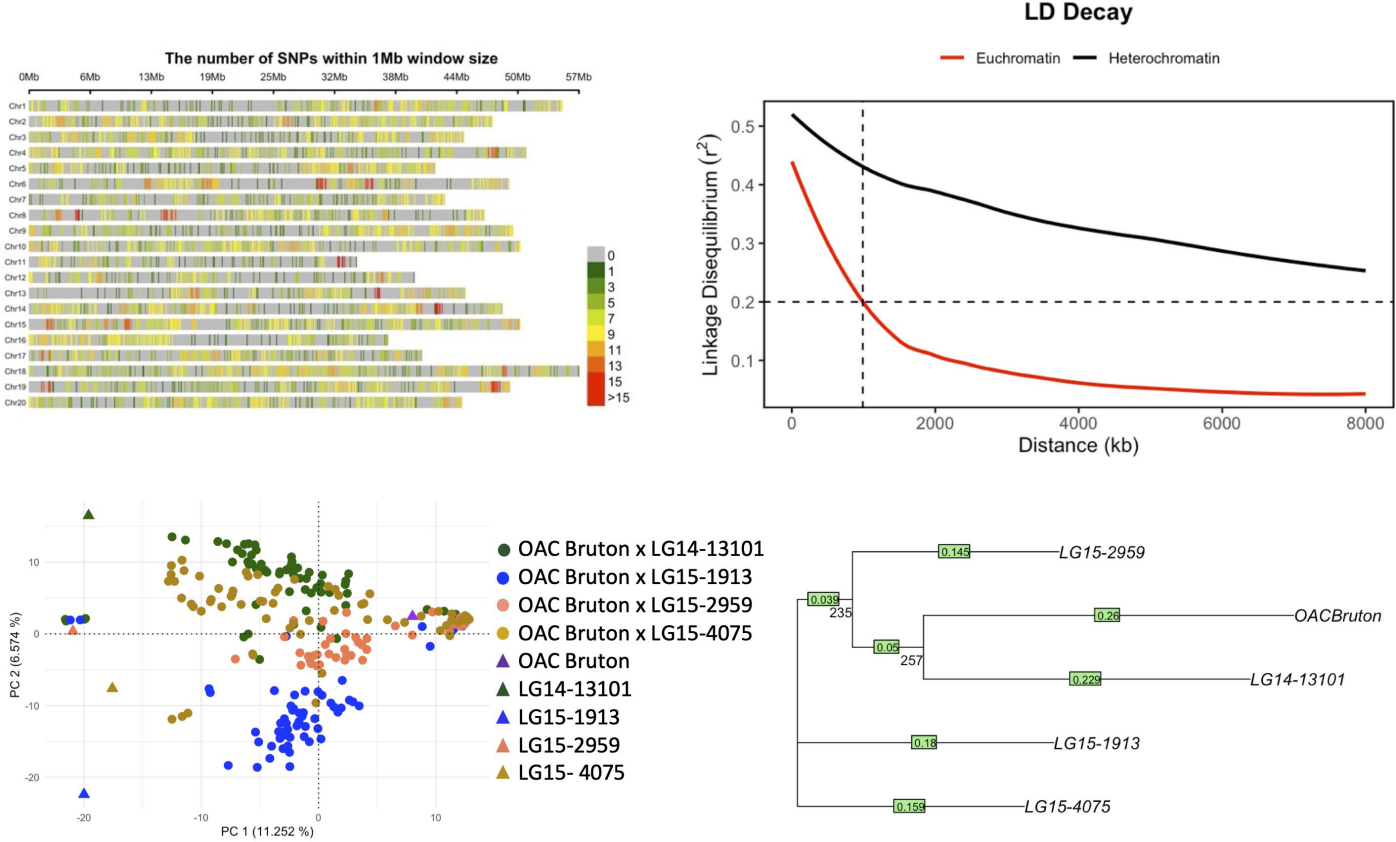
**(A)** Distribution of 5,213 single-nucleotide polymorphisms (SNPs) across 20 chromosomes in the NAM panel composed of four elite × exotic crosses (n = 253). **(B)** Linkage disequilibrium (LD) decay in heterochromatin and euchromatin regions where dashed line represents LD decay distance at the background r^2^ threshold of 0.2. **(C)** Principal component analysis (PCA) plot of first two principal components (PCs). **(D)** Neighbor-joining tree of parental lines OAC Bruton, LG14-13101, LG15-1913, LG15-2959, and LG15-4075. Bootstrap values (1,000 replicates) are shown below the branches.

The distribution of 5,213 SNPs across the 20 soybean chromosomes is shown in **Figure 2A**. Genome-wide estimate LD decayed to a baseline threshold of 0.2 r^2^ in 989 kb in euchromatin regions (**Figure 2B**). Based on the IBS neighbor-joining tree, LG14-13101 is most similar to OAC Bruton, while LG15-1913 and LG15-4075 are more genetically distant from OAC Bruton (**Figure 2C**). The PC analysis also captured the same patterns of genetic distance, where PC1 distinguished OAC Bruton from all exotic parents and PC2 distinguished LG15-4075 and LG15-1913 from the other three parents (**Figure 2D**). The top three PCs explained a total of 23.79% of the genomic variation (11.15%, 6.57%, and 6.07%).

### 3.3 Nested association mapping of agronomic traits

**Figure 3:**
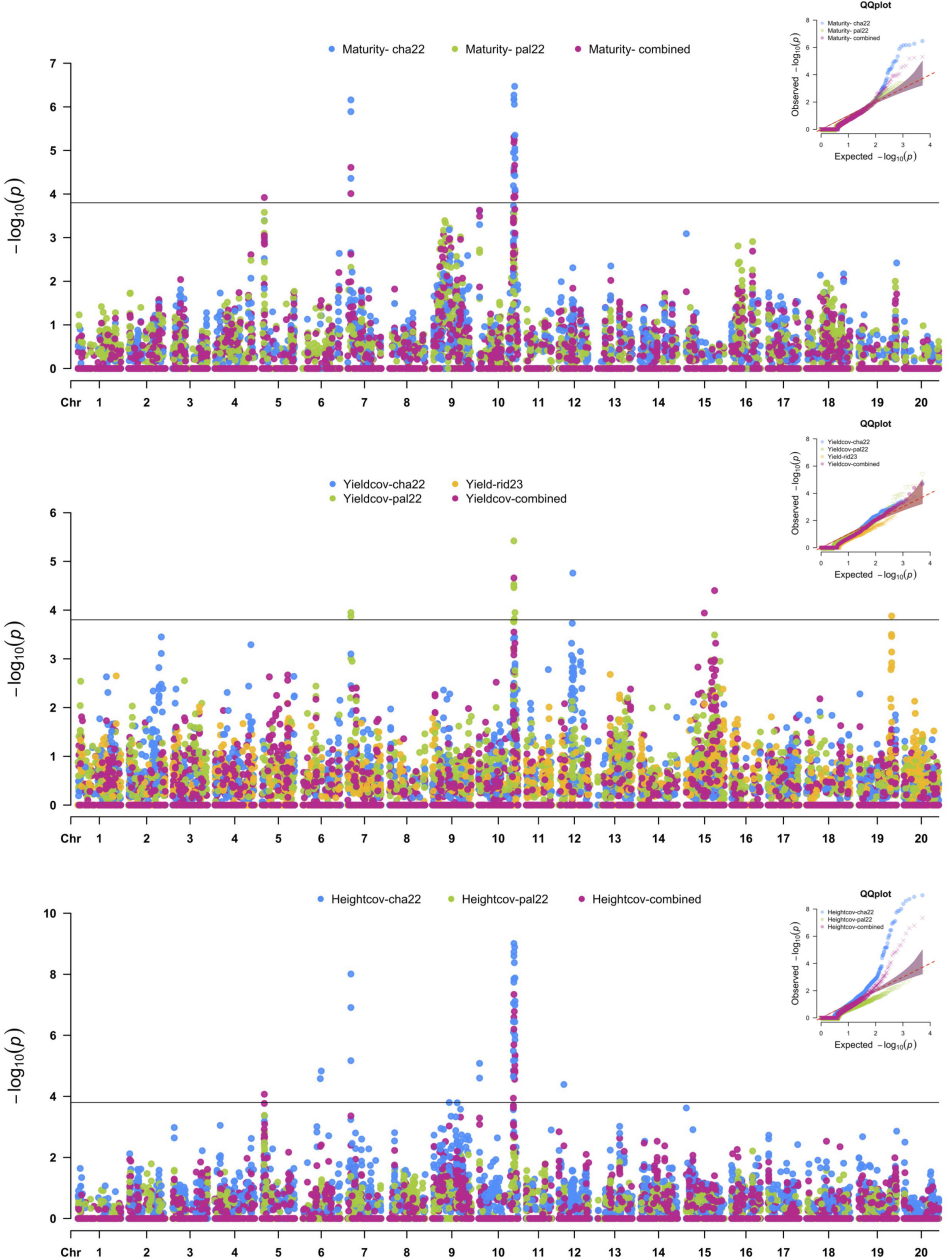
Manhattan plots of nested association mapping (NAM) analysis for maturity, seed yield, and height across three environments, Chatham 2022, Palmyra 2022, and Ridgetown 2023, as well as the combined environment analysis. Significant single-nucleotide polymorphisms (SNPs) were determined by the false discovery rate (FDR) 0.05 threshold (3.8 × 10^−5^) denoted by the horizontal line.

SNP markers that were significantly associated with the agronomic traits are summarized in **Figure 3** and **Table 3**. The SNP with the largest −log10(p-value) was used to define significant MTAs or genomic regions associated with the target traits. Additive allelic effects were estimated relative to the common parent (OAC Bruton), where the positive effect represents an increase in trait value when substituting the OAC Bruton allele with the respective exotic allele. Four SNPs within a 1,575-kb haplotype block on chromosome 7 (**Supplementary Figure 2**) and 24 SNPs located within a 3,000-kb haplotype block on chromosome 10 (**Supplementary Figure 3**) were significantly associated with maturity, seed yield, and plant height. On chromosome 7, the SNPs had an allelic effect ranging from −1.6 to −1.0 days on maturity, −208 to −168 kg/ha on seed yield, and −11.6 to −8.9 cm on plant height depending on the SNP and environment (**Table 3**). SNP S07_3993832 was mapped 109 kb upstream from the known *E11* maturity gene (*Glyma.07g048500*), which plays a role in early flowering and maturity under long days (Wang et al., 2019). On chromosome 10, the SNPs had allelic effects ranging from 1.3 to 2.0 days on maturity, 116 to 169 kg/ha on seed yield, and −2.8 to 4.5 cm on plant height depending on the SNP and environment (**Table 3**). The two leading SNPs, S10_45303725 and S10_45307266, co-located to the *E2* locus (*Glyma.10g221500*), a known regulator of flowering time and subsequently maturity in soybean (Watanabe et al., 2011).

**Table 3:** Summary of significant single-nucleotide polymorphisms (SNPs) from nested association mapping (NAM) of maturity, seed yield, and plant height across single and combined environment analyses (Chatham 2022, Palmyra 2022, and Ridgetown).

Environment	Chromosome	SNP	Alleles	MAF	−log10(p-val)	Allelic effect	Std.Dev
Maturity							
cha-22	10	S10_46228278	C/T	0.099	6.47	1.71	0.592
cha-22	10	S10_45307266	T/C	0.107	6.27	1.97	0.657
cha-22	10	S10_45303725	C/A	0.095	6.17	2.05	0.658
cha-22	10	S10_45322204	A/T	0.103	6.17	1.82	0.671
cha-22	7	S07_3993832	A/G	0.075	6.16	−1.58	1.116
cha-22	10	S10_45818758	G/A	0.107	6.06	2.06	0.943
cha-22	7	S07_3970904	A/T	0.087	5.89	−1.36	1.021
cha-22	10	S10_46703733	G/T	0.138	5.35	1.63	0.668
cha-22	10	S10_46457385	A/G	0.119	5.05	1.55	0.424
cha-22	10	S10_46497855	C/A	0.111	4.98	1.43	0.410
cha-22	10	S10_44738688	T/C	0.103	4.95	1.51	0.327
cha-22	10	S10_46750039	G/A	0.130	4.82	1.59	0.577
cha-22	10	S10_45246262	C/G	0.059	4.59	1.83	0.323
cha-22	10	S10_45245786	T/A	0.079	4.45	1.70	0.347
cha-22	10	S10_46578507	A/G	0.087	4.42	1.30	0.470
cha-22	7	S07_3975406	T/C	0.079	4.36	−1.36	0.650
cha-22	7	S07_3975419	C/T	0.079	4.36	−1.36	0.650
cha-22	10	S10_44623899	T/A	0.142	4.19	1.45	0.376
cha-22	10	S10_46707245	G/A	0.111	4.1	1.35	0.590
Combined	10	S10_45307266	T/C	0.107	5.31	1.72	0.574
Combined	10	S10_45818758	G/A	0.107	5.24	1.88	1.050
Combined	10	S10_45322204	A/T	0.103	5.18	1.58	0.715
Combined	10	S10_46228278	C/T	0.099	4.66	1.37	0.316
Combined	7	S07_3993832	A/G	0.075	4.61	−1.29	0.959
Combined	10	S10_45303725	C/A	0.095	4.52	1.63	0.511
Combined	10	S10_46750039	G/A	0.130	4.06	1.38	0.362
Combined	7	S07_3970904	A/T	0.087	4.01	−1.02	0.899
Combined	10	S10_45245786	T/A	0.079	3.94	1.50	0.554
Combined	10	S10_46703733	G/T	0.138	3.94	1.33	0.436
Combined	10	S10_46497855	C/A	0.111	3.93	1.21	0.320
Combined	5	S05_3017500	C/A	0.119	3.92	−1.69	0.722
Combined	10	S10_45246262	C/G	0.059	3.92	1.63	0.478
Seed yield							
cha-22	12	S12_17859607	C/T	0.051	4.76	−277.5	83.836
pal-22	10	S10_45245786	T/A	0.079	5.42	154.6	170.810
pal-22	10	S10_45303725	C/A	0.095	4.53	169.2	67.837
pal-22	10	S10_45246262	C/G	0.059	4.49	146.4	155.008
pal-22	10	S10_45322204	A/T	0.103	4.46	152.8	77.057
pal-22	7	S07_3970904	A/T	0.087	3.95	−186.3	101.967
pal-22	10	S10_46652617	A/C	0.091	3.95	148.1	46.918
pal-22	7	S07_3993832	A/G	0.075	3.87	−207.7	120.620
pal-22	10	S10_45818758	G/A	0.107	3.82	140.9	77.888
rid-23	19	S19_43110461	T/G	0.123	3.88	120.4	56.685
Combined	10	S10_45303725	C/A	0.095	4.66	116.4	18.449
Combined	15	S15_38087710	T/C	0.087	4.4	−94.3	91.542
Combined	15	S15_24978227	A/C	0.087	3.94	−92.5	71.776
Plant height							
cha-22	6	S06_23558630	G/A	0.091	4.58	6.54	23.882
cha-22	6	S06_24800605	T/C	0.079	4.83	6.12	22.048
cha-22	7	S07_3970904	A/T	0.087	6.91	−9.50	8.233
cha-22	7	S07_3975406	T/C	0.079	5.17	−8.88	6.669
cha-22	7	S07_3975419	C/T	0.079	5.17	−8.88	6.669
cha-22	7	S07_3993832	A/G	0.075	8.01	−11.59	9.415
cha-22	9	S09_21617193	T/C	0.130	3.8	−0.94	9.172
cha-22	10	S10_1281834	C/A	0.075	5.08	−4.14	8.751
cha-22	10	S10_1312881	G/A	0.079	4.6	−3.42	8.776
cha-22	10	S10_44550139	T/C	0.095	5.49	3.81	16.618
cha-22	10	S10_44550402	A/C	0.103	4.65	3.97	15.749
cha-22	10	S10_44570580	T/C	0.103	4.85	1.72	12.375
cha-22	10	S10_44623899	T/A	0.142	6.45	3.43	14.972
cha-22	10	S10_44653917	T/G	0.142	5.17	1.76	14.418
cha-22	10	S10_44724808	G/C	0.130	6.1	2.92	15.377
cha-22	10	S10_44738688	T/C	0.103	7.05	3.11	15.454
cha-22	10	S10_45245786	T/A	0.079	7.74	2.50	19.755
cha-22	10	S10_45246262	C/S	0.059	7.85	3.35	21.233
cha-22	10	S10_45303725	C/A	0.095	9.01	2.82	21.931
cha-22	10	S10_45307266	T/C	0.107	8.6	1.95	20.559
cha-22	10	S10_45322204	A/T	0.103	8.73	1.09	20.097
cha-22	10	S10_45818758	G/A	0.107	8.38	1.28	22.199
cha-22	10	S10_46228278	C/T	0.099	8.89	4.11	16.935
cha-22	10	S10_46457385	A/G	0.119	6.44	2.16	15.391
cha-22	10	S10_46497855	C/A	0.111	6	1.49	13.520
cha-22	10	S10_46578507	A/G	0.087	6.99	4.49	13.895
cha-22	10	S10_46652617	A/C	0.091	5.2	3.44	13.368
cha-22	10	S10_46703733	G/T	0.138	7.88	3.74	17.541
cha-22	10	S10_46707245	G/A	0.111	5.85	1.12	14.683
cha-22	10	S10_46750039	G/A	0.130	7.12	3.56	17.427
cha-22	12	S12_6392520	R/G	0.079	4.39	5.78	20.205
Combined	5	S05_3015627	A/T	0.091	4.07	4.66	3.437
Combined	5	S05_3015654	C/T	0.091	4.07	4.66	3.437
Combined	10	S10_44623899	T/A	0.142	3.94	−0.95	7.234
Combined	10	S10_45245786	T/A	0.079	5.69	−0.94	11.198
Combined	10	S10_45246262	C/G	0.059	4.83	−0.60	11.710
Combined	10	S10_45303725	C/A	0.095	6.2	−1.72	11.448
Combined	10	S10_45307266	T/C	0.107	6.59	−1.95	11.410
Combined	10	S10_45308689	A/G	0.032	4.7	−2.24	11.813
Combined	10	S10_45322204	A/T	0.103	7.34	−2.18	11.488
Combined	10	S10_45818758	G/A	0.107	6.78	−2.78	14.241
Combined	10	S10_46228278	C/T	0.099	4.56	−0.19	7.953
Combined	10	S10_46457385	A/G	0.119	5.02	−1.72	8.886
Combined	10	S10_46497855	C/A	0.111	4.85	−1.49	7.877
Combined	10	S10_46703733	G/T	0.138	5.33	−0.76	9.671
Combined	10	S10_46707245	G/A	0.111	4.8	−1.74	8.653
Combined	10	S10_46750039	G/A	0.130	5.35	−1.35	9.364

Allelic effects are relative to the common parent (OAC Bruton), indicating a positive or negative effect from substituting the OAC Bruton allele with an exotic allele.

MAF, minor allele frequency.

Additionally, SNP S05_3017500 was significantly associated with maturity in the combined environment analysis with an allelic effect of −1.7 days of reduction in maturity date but was not detected in any of the single environments. This SNP was mapped 246 kb upstream of previously detected QTLs for maturity in an early-season soybean population (Copley et al., 2018). The proposed candidate gene underlying this QTL, *Glyma.05g036300*, encodes spermidine/spermidine synthase, which is an enzyme that plays a role in embryo development and survival (**Table 4**).

**Table 4:** Summary of candidate genes of associated single-nucleotide polymorphisms (SNPs) for maturity, seed yield, and plant height.

Chr	Gene	Associated SNPs	Start	Stop	Functional annotation	Reference
Seed yield						
Gm10	*Glyma.10g221500*	S10_46707245, S10_45307266	45294735	45316121	Gigantea protein regulation of photoperiodism, flowering	(Watanabe et al., 2011)
Gm07	*Glyma.07g048500*	S07_3993832	4102968	4114174	LHY1/CCA1-like protein	(Wang et al., 2019
Gm12	*Glyma.12g137600*	S12_17859607	16399198	16400012	Suppressor of phyA-105 protein family (reproductive structure development)	
	*Glyma.12g139000*		16937219	16942610	Organic cation/carnitine transporter4 (transport)	
	*Glyma.12g139300*		17138889	17140028	Beta glucosidase 41 (carbohydrate metabolic process)	
	*Glyma.12g139500*		17166711	17167139	Beta glucosidase 41 (carbohydrate metabolic process)	
	*Glyma.12g139900*		17196729	17197567	Terpenoid biosynthetic process (cell differentiation, root growth and development)	
Gm15	*Glyma.15g217800*	S15_38087710	36667180	36670513	SWITCH1 (lipid metabolic process, multicellular organismal development)	
	*Glyma.15g218800*		37463406	37464616	BED zinc finger; hAT family dimerization domain (post-embryonic development)	
	*Glyma.15g219500*		38238490	38239720	Seven transmembrane MLO family protein (defense response, response to biotic stimulus)	
Gm19	*Glyma.19g161400*	S19_43110461	42212766	42214172	Photosystem II reaction center W protein (photosynthesis)	
	*Glyma.19g165400*		42633659	42635685	Sugar isomerase (SIS) family protein (carbohydrate metabolic process	
	*Glyma.19g179100*		43824144	43826915	Nucleotide-diphospho-sugar transferases superfamily protein (carbohydrate metabolic process)	
	*Glyma.19g186400*		44498441	44500784	Phosphatidyl inositol monophosphate 5 kinase (carbohydrate metabolic process)	
	*Glyma.19g159700*		42064879	42066838	emp24/gp25L/p24 family/GOLD family protein (transport)	
	*Glyma.19g168200*		42908207	42915046	Nuclear transport factor 2 (NTF2) family protein (transport)	
	*Glyma.19g176000*		43596780	43600042	ABC-2 type transporter family protein (transport)	
	*Glyma.19g181300*		44007407	44009765	Plasma membrane intrinsic protein 2A (transport)	
	*Glyma.19g186100*		44462011	44463438	Gamma tonoplast intrinsic protein (transport)	
	*Glyma.19g160400*		42123579	42125238	Flower development	
	*Glyma.19g160700*		42138179	42145544	Acetyltransferase (GNAT) family (flower development)	
	*Glyma.19g162500*		42327858	42329018	Zinc finger protein 11 (flower development)	
	*Glyma.19g170200*		43086883	43089910	RNA POLYMERASE-ASSOCIATED PROTEIN RTF1 HOMOLOG (flower development)	
	*Glyma.19g180300*		43905106	43907015	NAC-like, activated by AP3/PI (flower development)	
Maturity						
Gm10	*Glyma.10g221500*	S10_46707245, S10_45307266	45294735	45316121	Gigantea protein regulation of photoperiodism, flowering	(Watanabe et al., 2011)
Gm07	*Glyma.07g048500*	S07_3993832	4102968	4114174	LHY1/CCA1-like protein	(Wang et al., 2019
Gm05	*Glyma.05g036300*	S05_3017500	3189678	3192613	Spermidine synthase 1 (biosynthetic metabolism)	
Plant height						
Gm05	*Glyma.05g023200*	S05_3015627	2025044	2030348	Glucuronidase 2 (cell growth)	
	*Glyma.05g024200*		2108083	2109600	Eukaryotic elongation factor 5A-1 (xylem development)	
	*Glyma.05g025400*		2208393	2210545	Fucosyltransferase 1 (cell wall biogenesis)	
	*Glyma.05g025600*		2240341	2241955	Expansin A1 (cell wall organization)	
	*Glyma.05g030000*		2581123	2583418	Homeobox 1 (regulation of cell growth, development)	
	*Glyma.05g030100*		2591656	2599152	Leucine-rich repeat protein kinase family protein (cellulose biosynthesis process)	
	*Glyma.05g039100*		3465774	3468662	Leucine-rich repeat transmembrane protein kinase (epidermis development)	
	*Glyma.05g040400*		3610681	3617683	Tryptophan aminotransferase related 2 (indoleacetic acid biosynthetic process)	
Gm06	*Glyma.06g219800*	S06_24800605	24915597	24922978	WRKY transcription factor 64 ( photomorphogenesis, plant growth)	
	*Glyma.06g220100*		25365581	25365998	Myb domain protein 9 (cell differentiation)	
Gm12	*Glyma.12g074100*	S12_6392520	5543485	5564378	Phototropin 1 (phototropism, signal transduction)	
	*Glyma.12g076500*		5869107	5875332	GATA transcription factor 11 (cell differentiation)	
	*Glyma.12g081600*		6457418	6457513	Cytochrome b6f complex unit (photosynthesis)	
	*Glyma.12g075900*		5822268	5826790	Protein of unknown function (cell growth)	
	*Glyma.12g073900*		5508365	5522772	Pseudo-response regulator 7 (response to abiotic stimuli)	
	*Glyma.12g075800*		5813285	5820142	Homeobox-leucine zipper family protein (cell growth, differentiation)	
	*Glyma.12g076200*		5851956	5855787	Auxin response factor 10 (growth and development, signal transduction)	
	*Glyma.12g077100*		5940924	5942542	senescence-associated gene (SAG) 12 cyteine protease	
	*Glyma.12g079800*		6254464	6256886	Myb domain protein 36 (cell differentiation)	

Candidate genes were mined 989 kb upstream and downstream of associated SNPs.

Four additional SNPs on chromosomes 12, 15, and 19 were associated with SY across single environments. In Chatham 2022, a single SNP on chromosome 12 (S12_17859607) had an allelic effect of 277 kg/ha decrease in yield. Based on gene ontology (GO) enrichment analysis conducted in Soybase (Grant et al., 2010), the possible candidate genes listed in **Table 4** are involved in various biological processes such as cell membrane transport, cell differentiation, root growth and development, and carbohydrate metabolism. A single SNP on chromosome 19 was significantly associated with SY in Ridgetown 2023. SNP S19_43110461 had an allelic effect of 120 kg/ha increase in yield. There are 16 candidate genes involved in biological processes that could possibly yield related activities such as photosynthesis, carbohydrate metabolism, nutrient transport, and flower development (**Table 4**). Additionally, two SNPs on chromosome 15 located on separate haplotype blocks (**Supplementary Figure 4**) were significantly associated with SY in the combined environment analysis but were not detected in any of the single environments. SNP S15_38087710 and S15_24978227 had allelic effects of −94 and −92 kg/ha, respectively.

Six additional SNPs on chromosomes 5, 6, 9, and 12 were significantly associated with PH (**Table 3**). In the combined environment analysis, two SNPs located within a 667-kb haplotype block on chromosome 5 (**Supplementary Figure 5**) had an allelic effect of 4.66 cm on plant height. We found five candidate genes related to growth and development that could possibly be related to stem growth and plant height (**Table 4**).

The two SNPs on chromosome 6, S09_21617193 and S12_6392520, were also detected in a single environment (Chatham 2022). SNPs S06_23558630 and S06_24800605, 1,241 kb apart, had allelic effects of 6.5 and 6.1 cm on plant height, respectively. SNPs S09_21617193 and S12_6392520 had allelic effects of −0.9 and 5.8 cm on plant height, respectively.

### 3.4 Nested association mapping of seed composition traits

**Figure 4:**
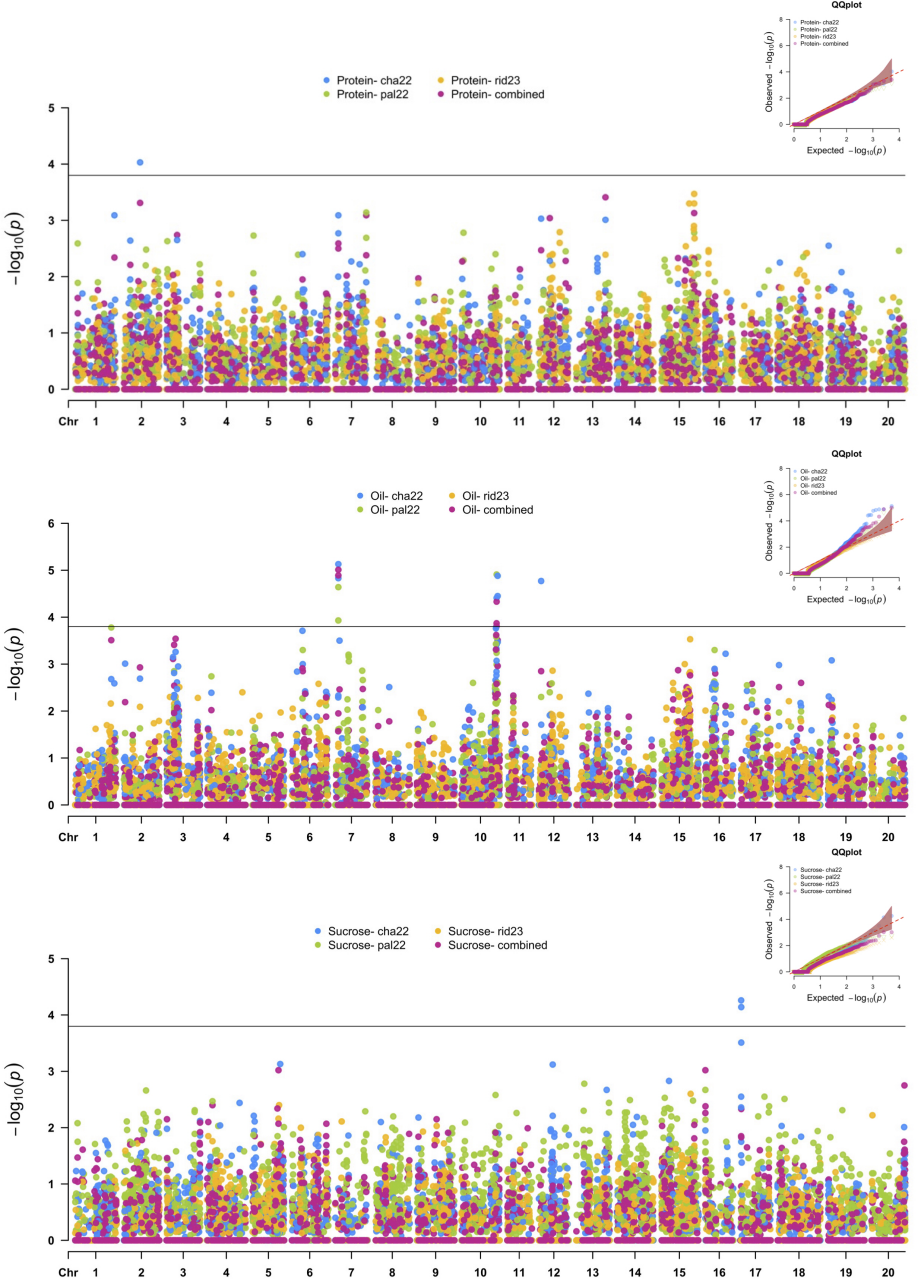
Manhattan plots of nested association mapping (NAM) analysis for protein, oil, and sucrose concentration across three environments, Chatham 2022, Palmyra 2022, and Ridgetown 2023, as well as the combined environment analysis. Significant single-nucleotide polymorphisms (SNPs) were determined by the false discovery rate (FDR) 0.05 threshold (3.8 × 10^−5^) denoted by the horizontal line.

Twelve SNPs in five distinct genomic regions were detected for PRO, OIL, and SUC across the 2022 environments, and no MTAs were detected in the Ridgetown 2023 environment (**Figure 4** and **Table 5**). For PRO, an environment-specific association was detected on chromosome 2 in Chatham 2022 with an allelic effect of −0.38% on protein concentration. For SUC, two SNPs were detected on chromosome 17 in Chatham 2022. These two SNPs were located within a 142-kb haplotype block (**Supplementary Figure 6**), and the leading SNP S17_1309007 had an allelic effect of 0.14% on sucrose concentration. We identified five possible candidate genes in this genomic region, which play a role in carbohydrate metabolism (**Table 6**). Two SNPs within the 1,575-kb haplotype block associated with agronomic traits on chromosome 7 (**Supplementary Figure 1**) and seven SNPs within two haplotype blocks on chromosome 10 (**Supplementary Figure 7**) were significantly associated with OIL across the 2022 environments and the combined environment analysis. On chromosome 7, the effect of the leading SNP ranged from 0.19% to 0.34% on oil concentration, and there were six candidate genes involved in lipid metabolism. On chromosome 10, the leading SNPs (S10_46750039 and S10_48180678) had effects ranging from −0.24% to −0.17% on oil concentration depending on the environment, and there were four candidate genes within the 989-kb region that encode enzymes involved in lipid metabolism (**Table 6**). Additionally, SNP S12_2995816 was significantly associated with OIL only in Chatham 2022, with an allelic effect of 0.49% increase in oil concentration.

**Table 5:** Summary of significant single-nucleotide polymorphisms (SNPs) from nested association mapping (NAM) of protein, oil, and sucrose concentration across single and combined environment analyses (Chatham 2022, Palmyra 2022, and Ridgetown).

Environment	Chromosome	SNP	Alleles	MAF	−log10(p-val)	Allelic effect	Std.Dev		
Protein concentration								Oil effect	Oil p-value
cha-22	2	S02_22011853	G/T	0.0435	4.03	−0.38	0.324	0.22	2.69
Oil concentration								Protein effect	Protein p-value
cha-22	7	S07_3993832	A/G	0.075	5.13	0.34	0.231	−0.30	3.09
cha-22	10	S10_48161072	T/C	0.063	4.88	−0.17	0.227	0.11	1.14
cha-22	10	S10_48161151	T/A	0.063	4.88	−0.17	0.227	0.11	1.14
cha-22	7	S07_3970904	A/T	0.087	4.83	0.30	0.251	−0.25	2.77
cha-22	12	S12_2995816	A/T	0.036	4.77	0.49	0.276	−0.32	3.03
cha-22	10	S10_48180671	C/A	0.087	4.45	−0.17	0.230	0.11	1.14
cha-22	10	S10_48180678	C/T	0.087	4.45	−0.17	0.230	−0.11	1.14
cha-22	10	S10_46750039	G/A	0.130	4.41	−0.24	0.130	−0.02	0
pal-22	10	S10_46750039	G/A	0.130	4.91	−0.22	0.045	−0.01	0
pal-22	7	S07_3993832	A/G	0.075	4.64	0.22	0.170	−0.17	1.45
pal-22	7	S07_3970904	A/T	0.087	3.93	0.19	0.153	−0.15	1.14
Combined	7	S07_3993832	A/G	0.075	5.01	0.24	0.166	−0.21	2.59
Combined	7	S07_3970904	A/T	0.087	4.89	0.21	0.176	−0.20	2.5
Combined	10	S10_46750039	G/A	0.130	4.33	−0.18	0.078	−0.01	0
Combined	10	S10_46703733	G/T	0.138	3.87	−0.17	0.061	−0.01	0
Combined	10	S10_46707245	G/A	0.111	3.81	−0.15	0.087	−0.02	0.4
Sucrose concentration									
cha-22	17	S17_1309007	T/G	0.095	4.26	0.14	0.122		
cha-22	17	S17_1451450	T/C	0.091	4.14	0.14	0.120		

Allelic effects are relative to the common parent (OAC Bruton), indicating a positive or negative effect from substituting the OAC Bruton allele with an exotic allele.

MAF, minor allele frequency.

**Table 6:** Summary of candidate genes of associated SNPs for protein, oil, and sucrose concentration.

Chr	Gene	Associated SNPs	Start	Stop	Functional annotation	Reference
Oil						
Gm10	*Glyma.10g37210*	S10_46750039	45239938	45245045	Pyruvate kinase activity (lipid metabolism)	(Watanabe et al., 2011)
	*Glyma.10g37820*		45713968	45,718,450	Alpha/beta-hydrolases superfamily protein (lipid metabolism)	
	*Glyma.10g40110*		47616449	47620842	Pyruvate kinase activity (lipid metabolism)	
Gm07	*Glyma.07g040500*	S07_3993832	3350340	3,356,964	LIPASE CLASS 3 FAMILY PROTEIN (lipid metabolism)	
	*Glyma.07g044100*		3667352	3,670,363	Carboxylesterase (lipid metabolism)	
	*Glyma.07g044200*		3675687	3,681,153	Carboxylesterase (lipid metabolism)	
	*Glyma.07g047400*		3977971	3,979,901	Protein of unknown function (lipid metabolism)	
	*Glyma.07g056800*		5039444	5,043,257	Galactolipase (lipid metabolism)	
	*Glyma.07g060500*		5378706	5,383,359	α-l-Fucosidase (lipid metabolism)	
Sucrose						
Gm17	*Glyma.17g003400*	S17_1309007, S17_1451450	370577	373210	Glycosyl hydrolase family 9 (carbohydrate metabolism)	
	*Glyma.17g006000*		551560	554178	5′-AMP-activated protein kinase beta-2 subunit protein (carbohydrate metabolism, cellular response to nitrogen levels)	
	*Glyma.17g012500*		965810	969000	*O*-Glycosyl hydrolases family 17 protein (carbohydrate metabolism)	
	*Glyma.17g029700*		1813337	1821780	Pectin lyase-like superfamily protein ((carbohydrate metabolism)	
	*Glyma.17g024700*		1813337	1821780	AMP-ACTIVATED PROTEIN KINASE, GAMMA REGULATORY SUBUNIT (regulatory component of carbohydrate metabolism)	
Protein						
Gm02	*Glyma.02g162300*	S02_22011853	21052064	21076949	Protein phosphatase 2C ( PP2C) family protein (response to biotic and abiotic stimuli)	
	*Glyma.02g163200*		21700163	21724365	P-loop containing nucleoside triphosphate hydrolases superfamily protein (transmembrane transport)	
	*Glyma.02g163100*		21664178	21664690	Protein of unknown function	
	*Glyma.02g162700*		21504898	21511492	Purple acid phosphatase 29 (nutrient acquisition and recycling)	
	*Glyma.02g164100*		22649058	22653051	4′-Phosphopantetheinyl transferase superfamily (lysine biosynthetic process via aminoadipic acid)	

Candidate genes were mined 989 kb upstream and downstream of associated SNP.

SNP, single-nucleotide polymorphism.

## 4 Discussion

The genetic distance between the parental lines and the results of PC analyses of the population suggested a discernible population structure within the population, attributed to the genetic background of the exotic parents. Among these, the exotic parent LG14-13101 was found to be most similar to the Canadian parent, OAC Bruton. This is likely due to the high percentage of elite parentage in the parental line LG14-13101, resulting from the backcrossing scheme used in its development. In this population, LD decay reached an r^2^ threshold of 0.2 within 989 kb in euchromatin regions. LD decay rates vary across populations and can be influenced by factors such as selection history, genetic diversity, and population stratification (Song et al., 2015). Hyten et al. (2007) reported varying LD decay rates: within 100 kb in *Glycine soja* and between 90 and 574 kb in Asian landraces and North American cultivars. The observed slower decay in our population, which is defined by four bi-parental crosses and five parental haplotype lines, is expected when compared to natural populations. Beche et al. (2020) also reported sustained LD in a NAM panel of three *G. soja* × adapted cross populations, where LD decayed to its half-life (r^2^ = 0.44) in 619.5 kb.

The primary aim of this research was to identify exotic genomic regions that contribute to yield, agronomic, and seed composition traits and would therefore be potential candidates for introgression into the Canadian soybean breeding programs. To achieve this, a NAM population was constructed with cross combinations of four high-yielding exotic parents and OAC Bruton, which possesses fixed North American alleles for yield and seed composition traits. GWAS revealed both positive alleles from the adapted parent and the exotic parents, although OAC Bruton, as expected, contributed a higher frequency of beneficial alleles for the target traits (**Tables 3**, **5**).

GWAS of agronomic traits in this NAM population identified both previously known and novel genomic regions underlying seed yield. The two most significant MTAs were the *E2* locus on chromosome 10, associated with maturity, yield, and plant height, and the E11 locus on chromosome 7, associated with maturity, yield, and plant height as well as oil concentration. In soybean, the *E2* locus (*Glyma.10g221500*) is one of the major genes controlling flowering through suppression of photoperiod response genes *GmFT2a* and *GmFT5a* (Watanabe et al., 2011). Notably, all exotic parents contributed the positive allele, which delayed maturity by 1.6 days and increased yield by 166 kg/ha in the combined analysis, while OAC Bruton contributed the early maturing allele. This genomic region explained 19% of the variation in maturity and 28% of the variation in seed yield (**Supplementary Figure 8**). The coupled effects of later maturity and yield increase are not surprising for a maturity QTL, as a longer growth period allows for growth and biomass conversion. Fang et al. (2017) and Diers et al. (2018) previously detected co-localization of maturity, yield height, and lodging at the *E2* locus, suggesting possible pleiotropism or tightly linked genes. In general, allelic variation in flowering and maturity could be exploited as a strategy for yield improvement, as delayed maturity is positively correlated with yield. Previous diversity analysis of the University of Guelph soybean germplasm revealed the early maturity *e2* allele was nearly fixed in the breeding germplasm (Bruce et al., 2019b). Therefore, introgression of the exotic allele may be useful in breeding strategies for yield improvement in Canadian germplasm, particularly in the context of a changing climate and longer growing seasons.

On chromosome 7, three out of four exotic parents possessed an unfavorable allele for yield, which shortened maturity by 1.3 days and increased oil concentration by 0.24%. In contrast, LG14-13101 and OAC Bruton possessed a favorable allele that delayed maturity and decreased oil. This genomic region explained 9% and 18% of the variation in maturity and oil, respectively (**Supplementary Figure 9**). Several novel exotic-based yield MTAs were also identified on chromosomes 12, 15, and 19. However, the presence of environment-specific associations for these SNPs suggests that the alleles may confer advantages only under specific environmental conditions. Additional study is recommended to confirm the yield-related role of these genomic regions. These significant SNPs should be further investigated and characterized through additional mapping studies using larger populations.

## 5 Conclusion

In this study, we detected seven unique genomic regions for agronomic traits on chromosomes 5, 6, 7, 9,10,12,15, and 19 and four regions associated with seed composition traits, containing both positive and negative alleles from the exotic parents. Notably, key loci on chromosomes 10 and 7 played significant roles in controlling maturity, yield, plant height, and oil concentration. The results of this study indicate the benefits of incorporating exotic germplasm for a successful introduction of novel diversity for maturity and yield into an elite food-grade soybean background, thereby providing a pathway to enhance genetic diversity and achieve sustainable yield improvements in food-grade soybean. Additionally, we identified several environment-specific MTAs for yield, demonstrating the complexity and importance of understanding genotype-by-environment interactions in breeding programs. Successful utilization of MTAs in selection depends on several factors, including the magnitude of the allelic effect, the accuracy of its mapped location, and the stability of its effect across diverse environments and targeted genetic backgrounds (Kage et al., 2016; Moose and Mumm, 2008). Therefore, before incorporating exotic-derived variants identified in this study into a marker-assisted selection (MAS) breeding framework, particularly when applying them to different germplasm beyond those of the University of Guelph, it is advisable to validate these variants across different Canadian-adapted genetic backgrounds. Additionally, further environmental testing is recommended to confirm their sustainable effects and to fully understand the environment-specific advantages they may provide.

## Data Availability

The datasets analyzed for this study can be found in the Figshare repository https://doi.org/10.6084/m9.figshare.26871199.v1.
