# Supplementary figures and images for: Genome-wide association mapping in exotic × Canadian elite crosses: mining beneficial alleles for agronomic and seed composition traits in soybean
Source: Front Plant Sci. 2024 Nov 14;15:1490767. doi: 10.3389/fpls.2024.1490767 (PMC11602288; doi:10.3389/fpls.2024.1490767)

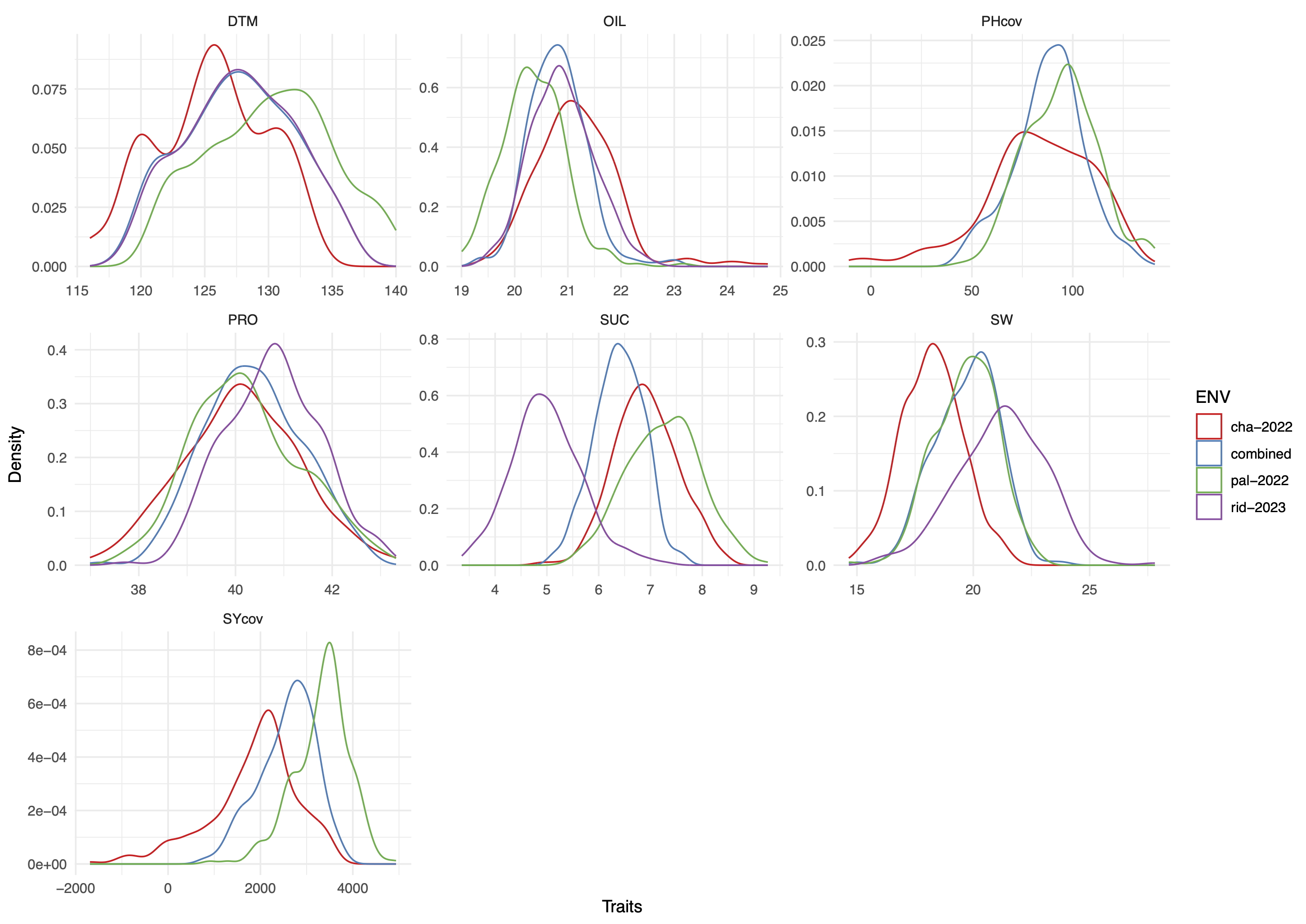

Supplement: Supplementary file 1 [file Image1.jpg]

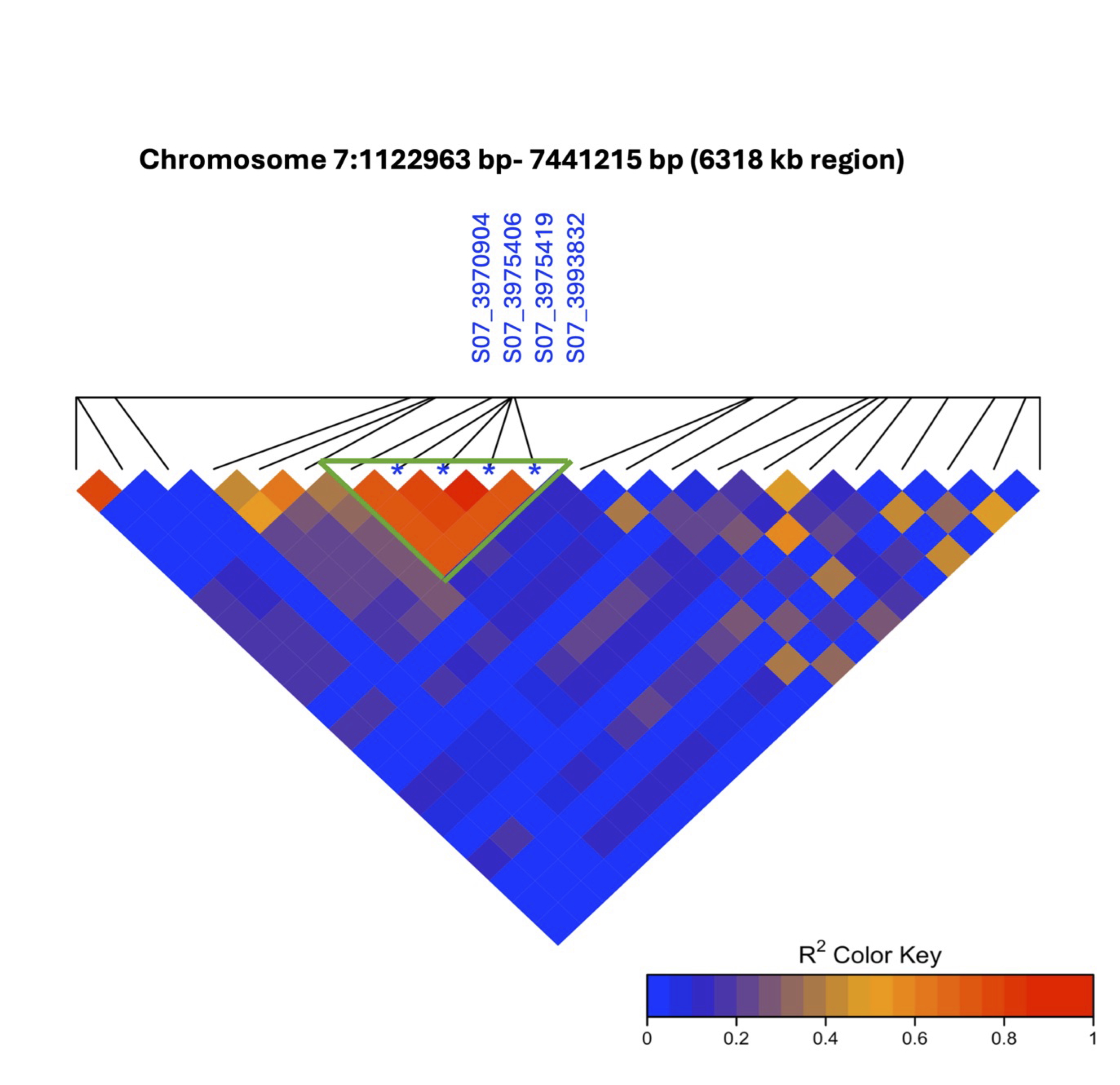

Supplement: Supplementary file 2 [file Image2.jpg]

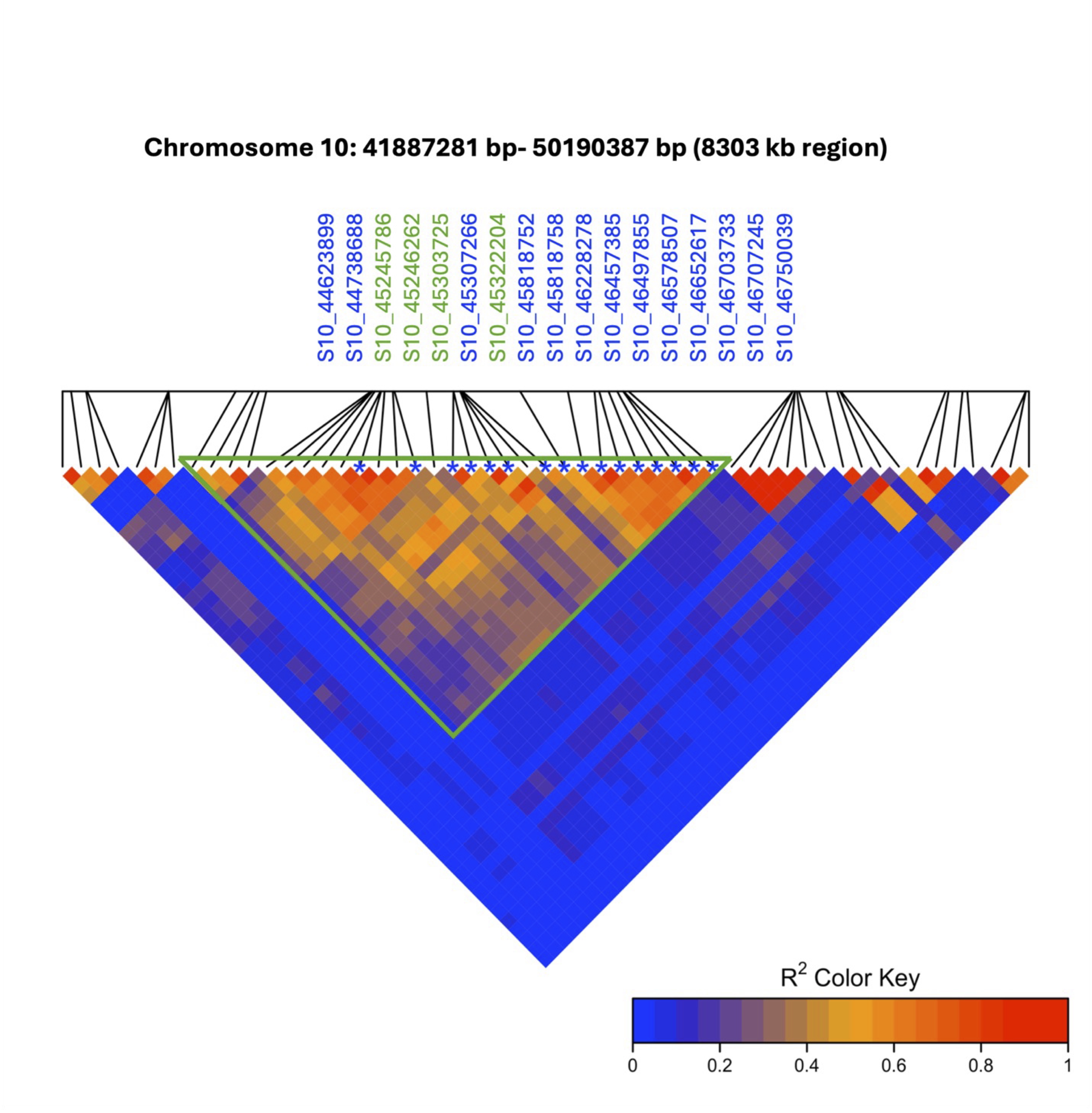

Supplement: Supplementary file 3 [file Image3.jpg]

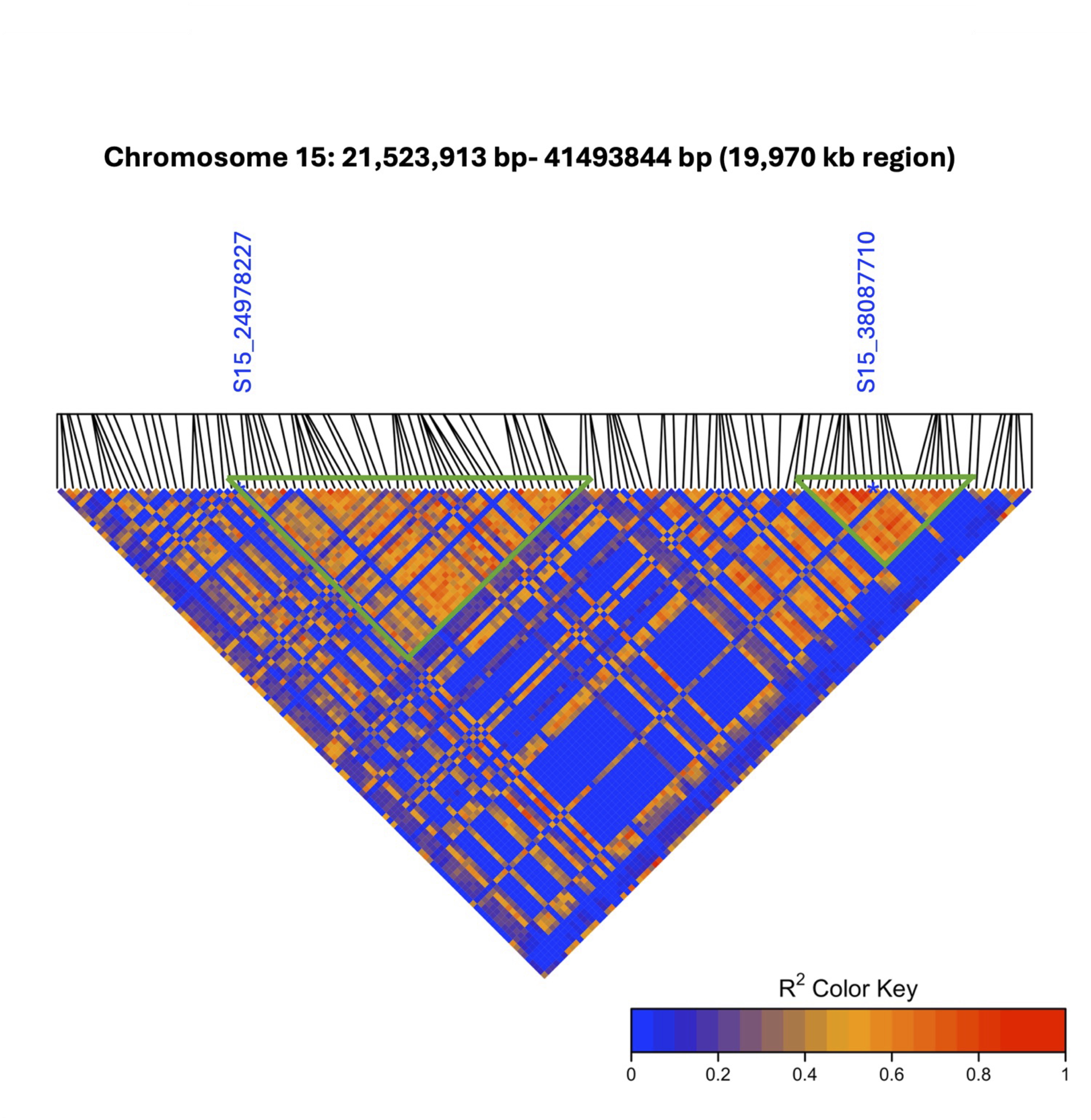

Supplement: Supplementary file 4 [file Image4.jpg]

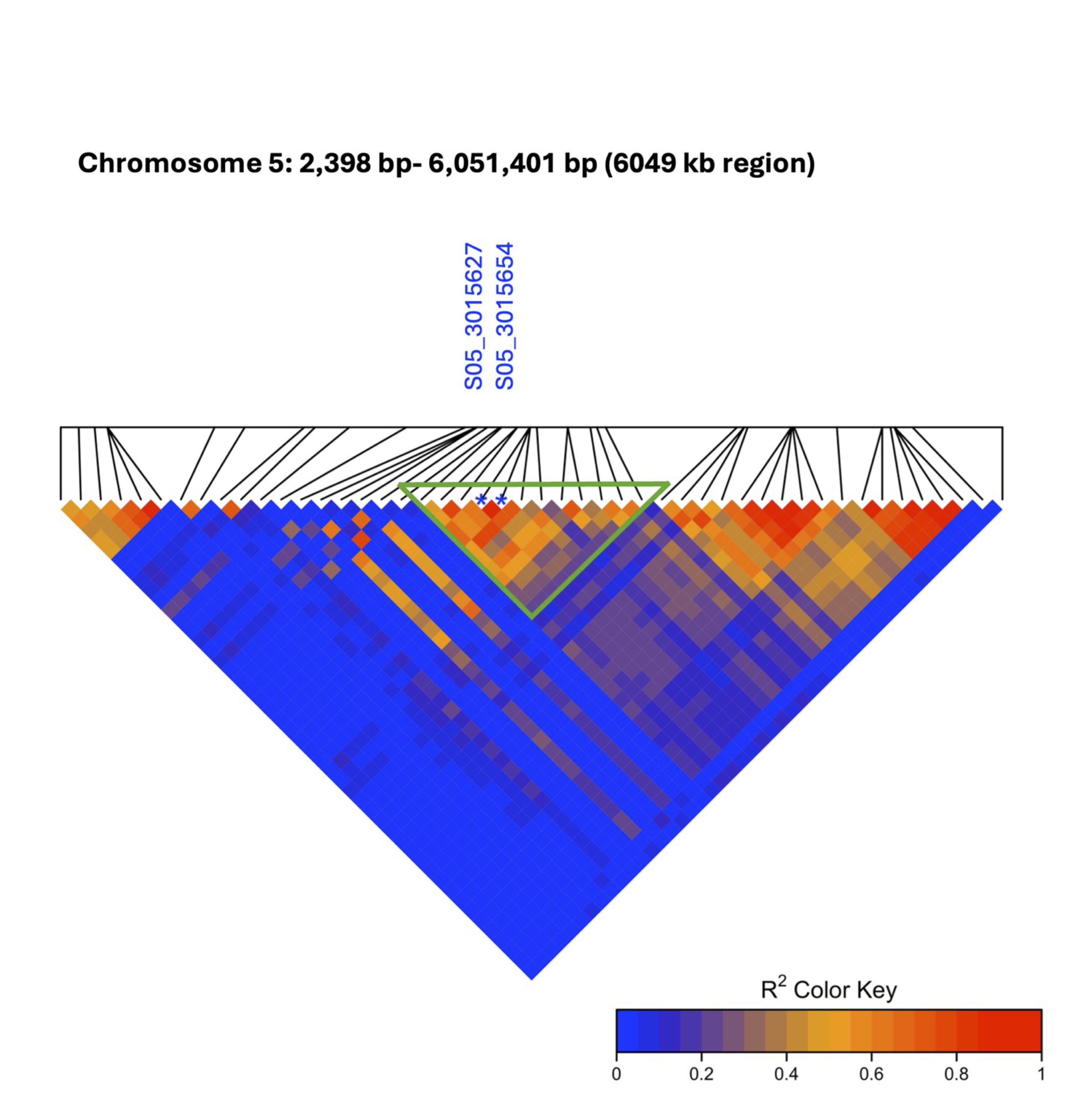

Supplement: Supplementary file 5 [file Image5.jpg]

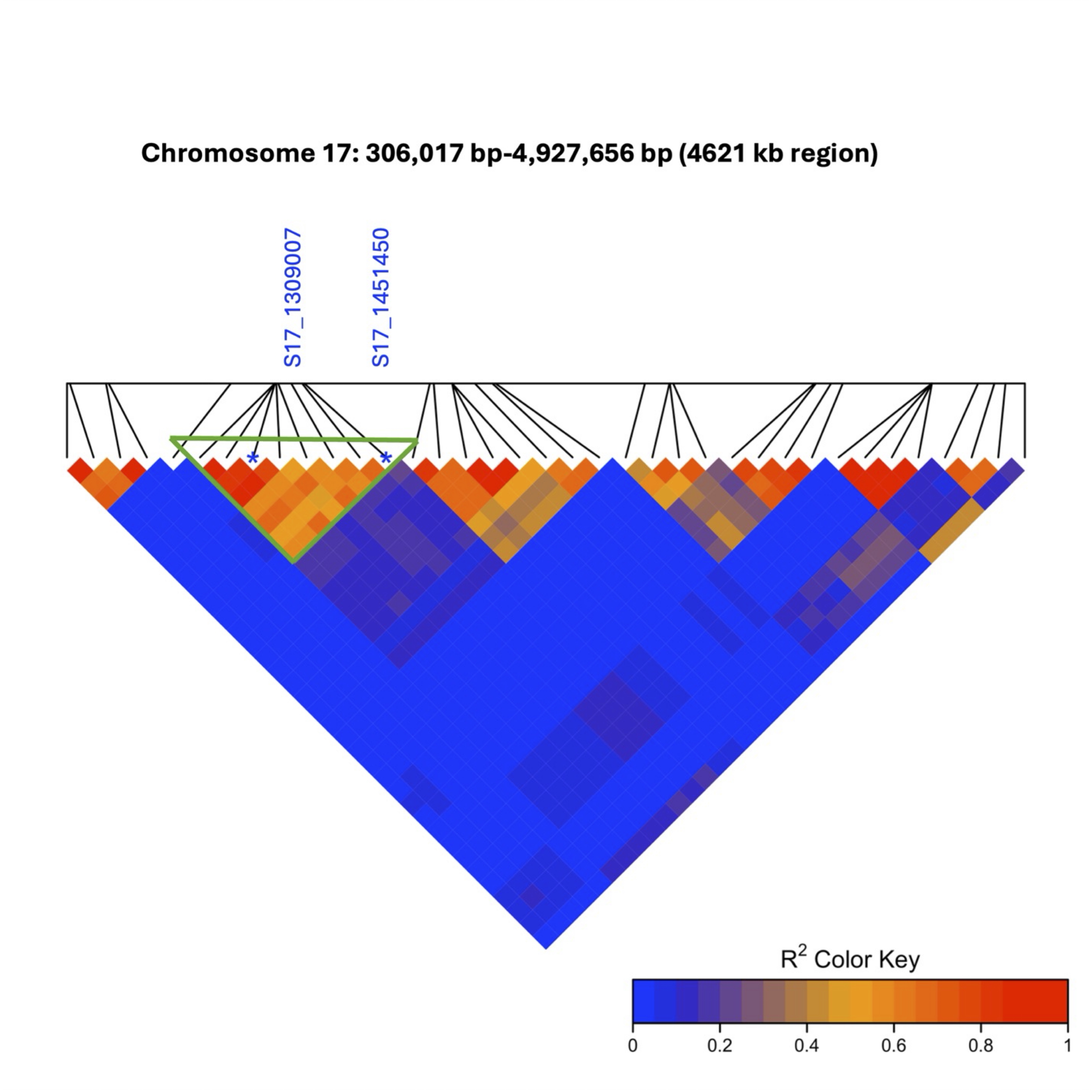

Supplement: Supplementary file 6 [file Image6.jpg]

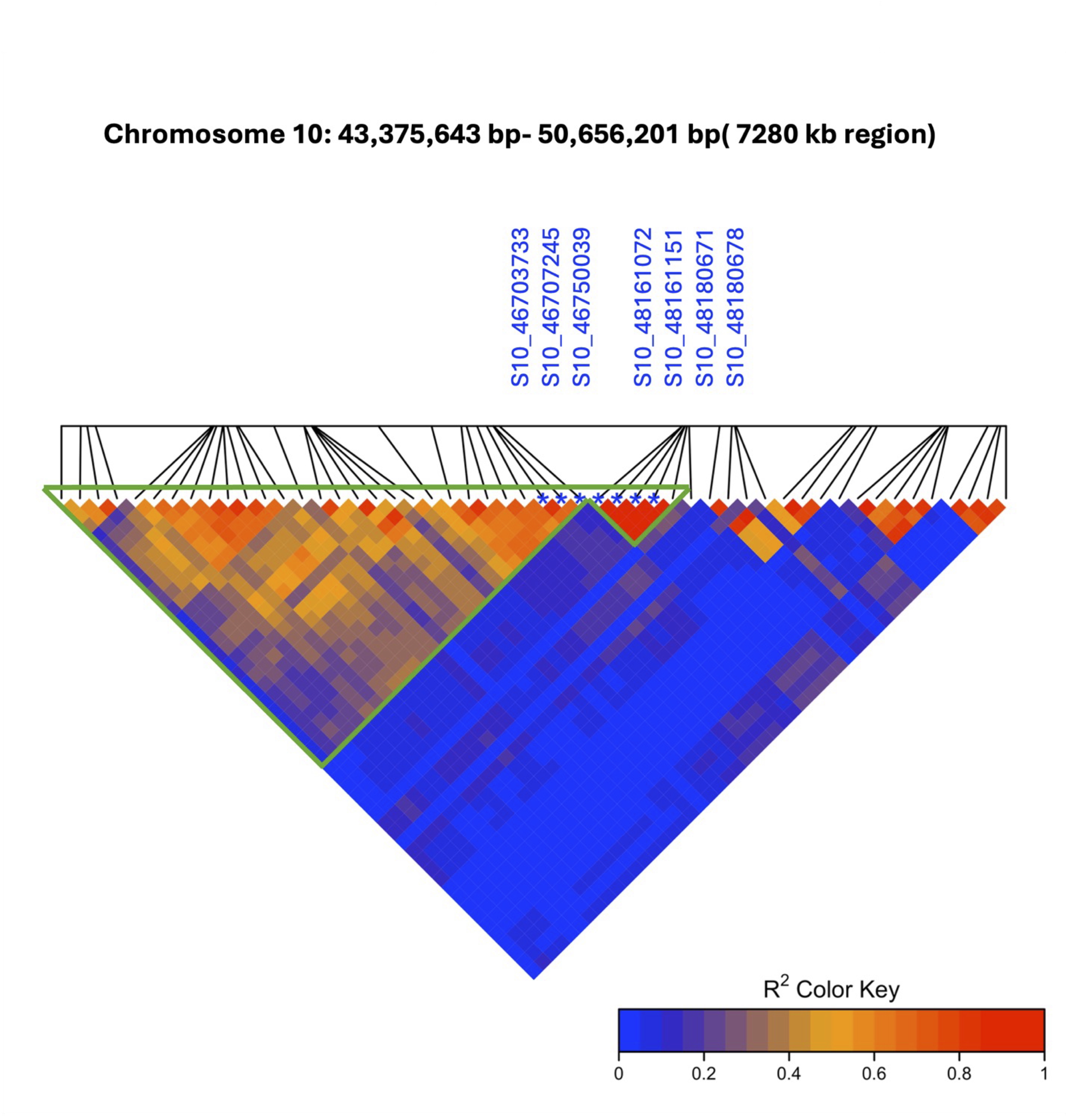

Supplement: Supplementary file 7 [file Image7.jpg]

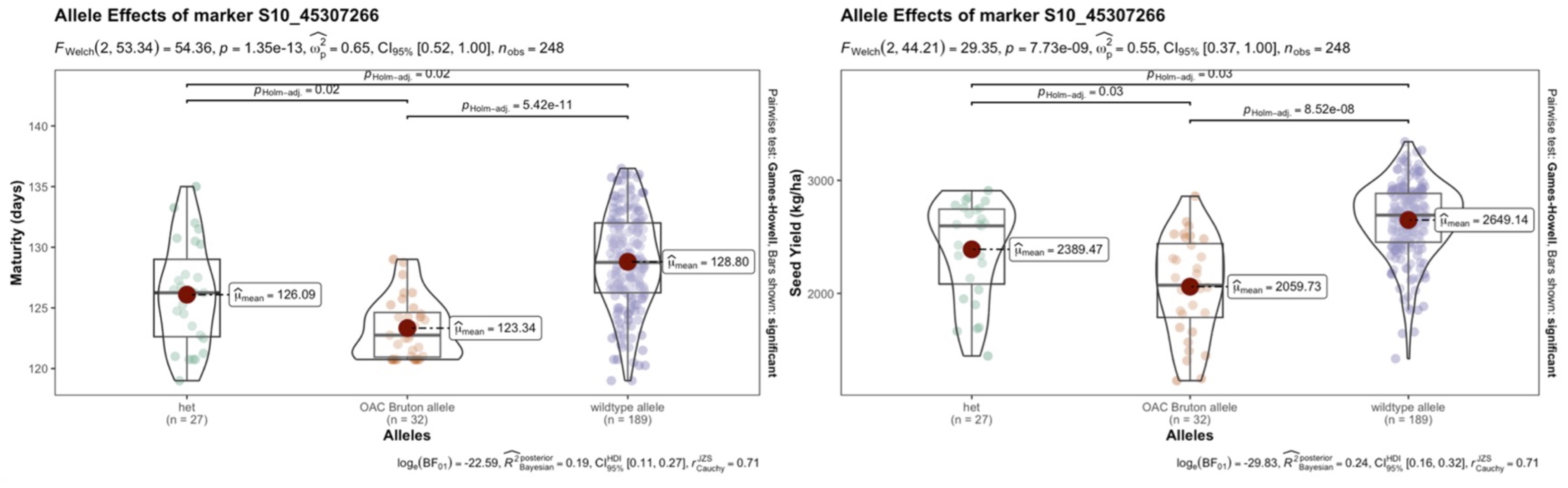

Supplement: Supplementary file 8 [file Image8.jpg]

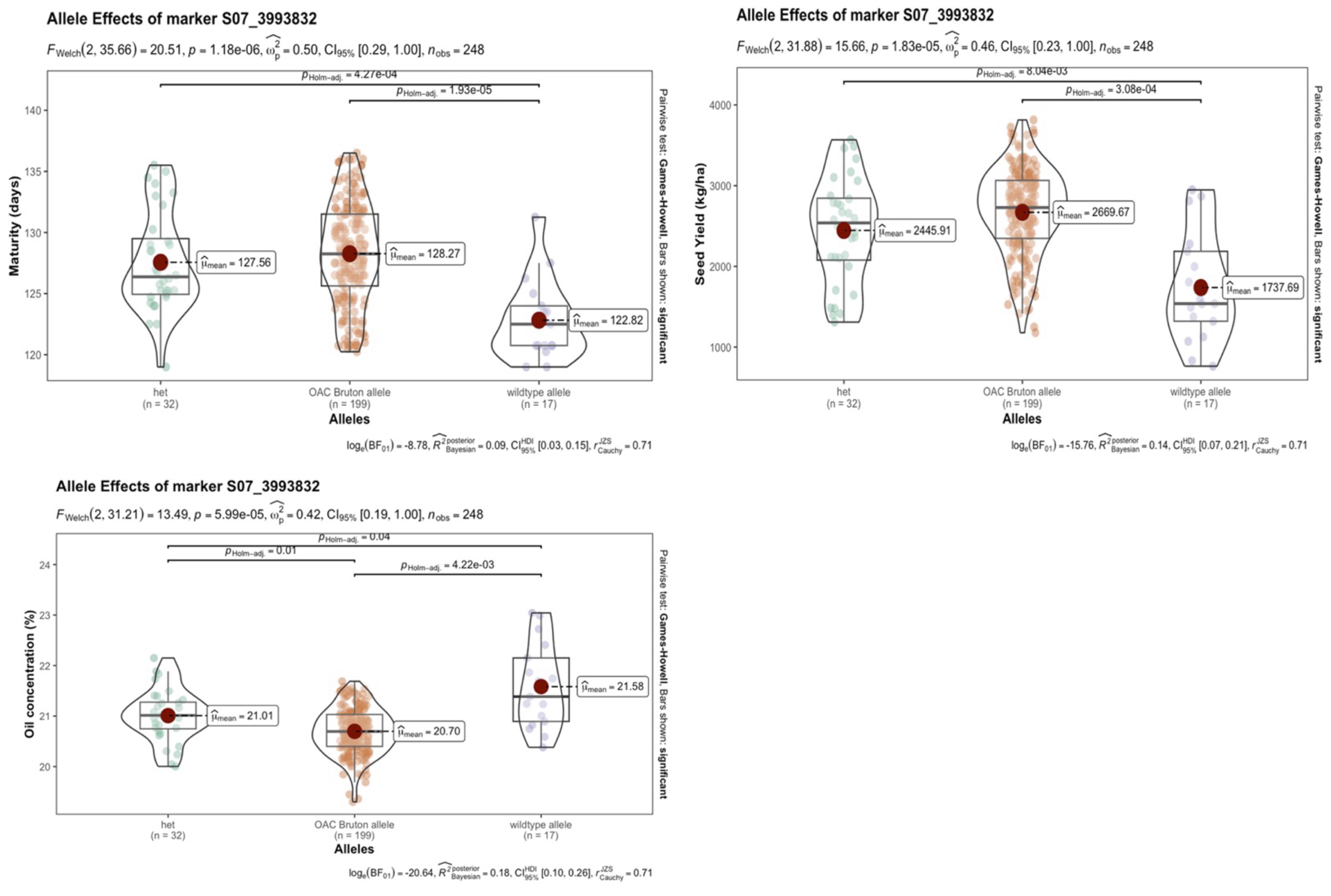

Supplement: Supplementary file 9 [file Image9.jpg]

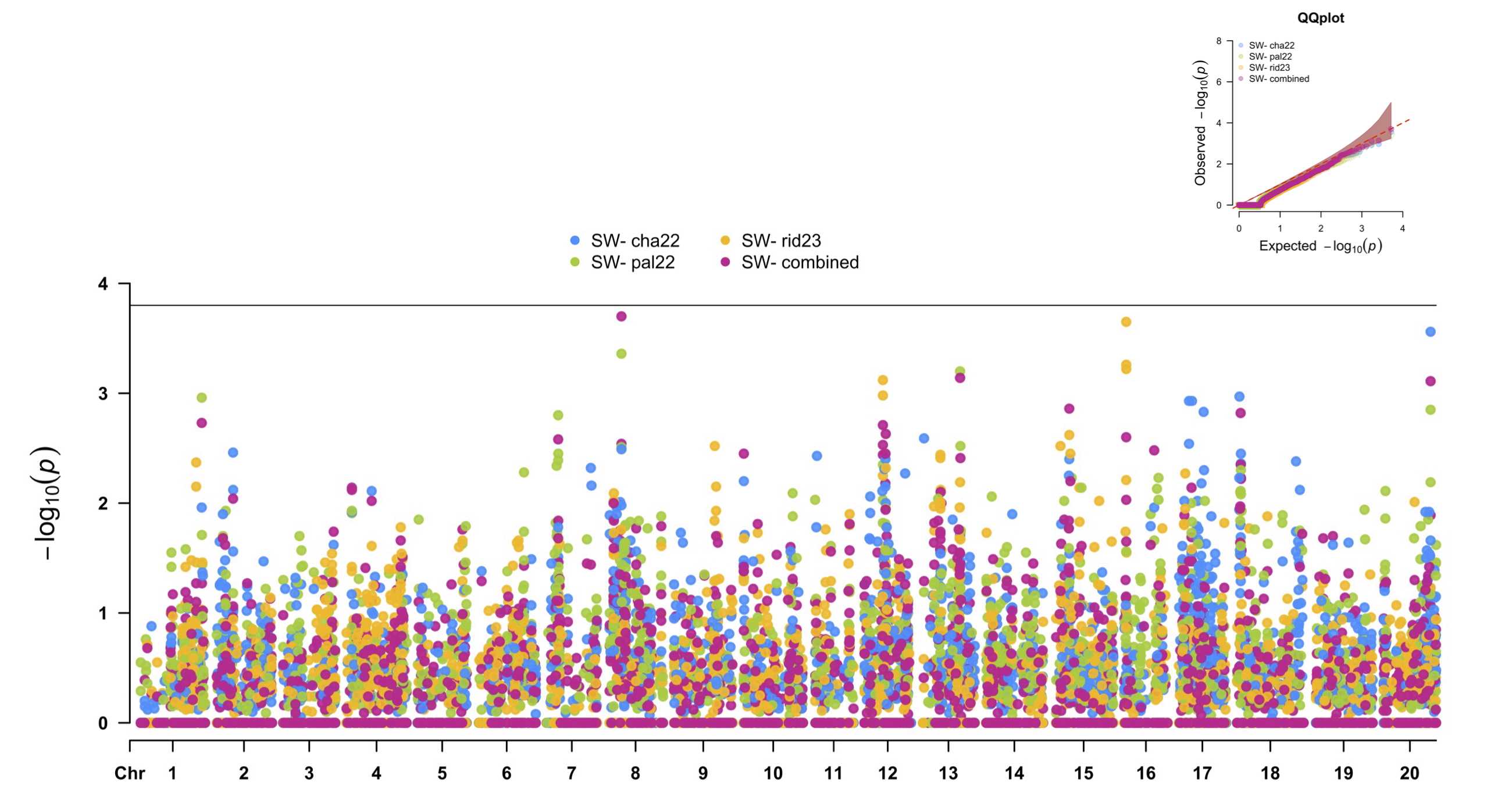

Supplement: Supplementary file 10 [file Image10.jpg]
